# Development and validation of bleeding prediction model for percutaneous liver biopsy in children

**DOI:** 10.1186/s12887-025-06341-w

**Published:** 2025-12-08

**Authors:** Yuyan Huang, Yiwen Zhou, Xiaofeng Xu, Junmei Jiang, Zhaoyang Gou, Yi Lu, Xinbao Xie, Jianshe Wang, Zhuowen Yu

**Affiliations:** Department of Hepatology, Children’s Hospital of Fudan University, 399 Wanyuan Road, Shanghai, Minhang District 201102 China

**Keywords:** Prediction model, Percutaneous liver biopsy, Children

## Abstract

**Objective:**

To evaluate the current status and factors influencing the occurrence of percutaneous liver biopsy bleeding in children through a retrospective study, and to develop and validate a risk prediction model to reduce the incidence of percutaneous liver biopsy bleeding in children.

**Methods:**

From the hospital's electronic medical record system, clinical data of the study subjects were obtained during their hospitalization. Continuous variables were described using the median (interquartile range), while categorical variables were described using frequencies, proportions, and rates. Feature variables were screened using Lasso regression, and the data were divided into training and validation sets in a 7:3 ratio. Variables with statistically significant differences were included in a binary logistic regression model, and a risk prediction model was constructed using stepwise bidirectional regression. The model was visualized using a nomogram and internally validated. The ROC curve was used to assess the model's discriminative ability, the calibration curve to evaluate its calibration, and the decision curve analysis to assess its clinical decision-making capability.

**Results:**

The incidence of bleeding in this study was 13.3%, most of which were minor and did not cause serious complications. Variables with meaningful Lasso regression coefficients were included in the multivariate logistic regression analysis, and the stepwise bidirectional regression ultimately yielded seven independent influencing factors: Pre-Corticosteroid, Post Liver Transplantation, Needle Depth, ALT, PT, PLT, and GPR. These factors will be used to construct a prediction model for percutaneous liver biopsy bleeding in children. In this study, the training set AUC was 0.720, with a 95% CI of 0.675-0.765, and the validation set AUC was 0.700, with a 95% CI of 0.633-0.767.

**Conclusion:**

This study created and internally tested a bleeding prediction model for children undergoing percutaneous liver biopsy, demonstrating moderate discriminative ability. Additional optimization and external validation are necessary. Expanding research with larger, multi-center datasets is crucial to enhancing the model's predictive accuracy and clinical applicability.

**Supplementary Information:**

The online version contains supplementary material available at 10.1186/s12887-025-06341-w.

Liver biopsy is the gold standard for diagnosis, prognosis and assessment of response to treatment in liver diseases [1], of which percutaneous liver biopsy is the most common type, possessing the advantages of being less invasive and more cost-effective [2]. Studies have shown [3] that the complication rate of percutaneous liver biopsy in pediatric patients ranges from 0 to 14%, with the majority of complications occurring within 4 h of liver biopsy, including pain, bleeding, infection, bile leakage, arteriovenous fistula, and hemopneumothorax. Bleeding is the most common complication in the all-age population, with the incidence of severe bleeding reported to be 0.1–4.6% and minor bleeding up to 10.9% according to available studies [1]. Some studies have shown that the incidence of bleeding after percutaneous liver biopsy is higher in children than in adults, up to 4.6%−18% [4]. Severe bleeding after percutaneous liver biopsy is the leading cause of postoperative mortality in children, and prospective prediction of the risk of bleeding in children undergoing percutaneous liver biopsy may provide theoretical guidance for clinical practice, improve diagnostic and treatment decisions and outcomes, and prevent or minimize the incidence of bleeding, thereby improving the cost-effectiveness of healthcare services.

Most of the current studies on percutaneous liver biopsy focus on the reliability and safety of ultrasound-guided percutaneous liver biopsy, as well as the incidence of bleeding complications [5–7], and some of the studies have explored the influencing factors of bleeding in percutaneous liver biopsy [1, 8]. However, the data are limited, and most of them present the correlation between a single risk factor and bleeding from liver puncture. Due to the complexity of the surgical procedure, children are often affected by multiple factors and cannot be judged using a single factor. Most of the existing prediction models focus on percutaneous liver biopsy associated with non-alcoholic cirrhosis. To our knowledge, there is no existing relevant prediction model to assess and predict the risk of bleeding from percutaneous liver biopsy in children. Therefore, we propose conducting a single-center retrospective study to evaluate the current status of bleeding complications following percutaneous liver biopsy in children and identify influencing factors. Additionally, we aim to develop and validate a risk prediction model to reduce the incidence of bleeding complications following percutaneous liver biopsy in children.

## Methods

### Participant

A single-center retrospective study of 1665 children with bleeding from percutaneous liver biopsy admitted to the Children’s Hospital of Fudan University from July 2017 to August 2023 was conducted.

### Inclusion criteria

(1) < 18 years old; (2)All data and information records are of high integrity.

### Exclusion criteria

(1) Those who failed to complete percutaneous liver biopsy successfully for any reason; (2) If the same patient was biopsied several times at different times, only the variable information of the first biopsy was included, and all the re-biopsies were excluded; (3)Those who already had a bleeding state before the puncture.

### Procedure and main steps of percutaneous liver biopsy

Ultrasound liver localization was performed on admission, and the recommended puncture points were marked. Tests such as a blood count and coagulation function were also performed. Before the puncture, the child’s vital signs were recorded. The clinician will decide whether or not to inject anesthetics intravenously according to the child’s age and compliance, and the most commonly used intravenous anesthetic in our center is “Liyuexi (midazolam injection)”. During the puncture, the children’s hands were raised above their heads and placed behind the head to maintain a comfortable position. Local anesthesia was administered by subcutaneous injection of 5% lidocaine at the ultrasonically located puncture point. The puncture needle was a semi-automatic, disposable, cutting type. After a puncture, the hole of the puncture needle was covered with sterile gauze, and local pressure was applied for twenty minutes. The obtained liver tissue specimen was placed in a vial specimen bottle of 10% formalin solution and immediately sent to the department of pathology for examination. The child will be asked to keep resting in bed for 4 h. The respiration, pulse, and blood pressure should be monitored once every 15 min in the first hour, once every 30 min in the following 2 h, and then once every hour until 6 h after the professional ultrasound teacher examines to make sure that there is no special bleeding situation.

### Devices

Color Doppler ultrasound diagnostic instrument (GE, Versana Premier SPt, China), convex array probe, linear array probe, frequency 4–12 MHz; Automatic non-coaxial biopsy gun system (16 G, 18 G disposable biopsy needle, automatic biopsy puncture gun Bard Magnum1522, Bard Medical Technology, USA).

### Data collection

Clinical data for all subjects during hospitalization were obtained from the hospital’s electronic medical record system (Fugao). This data included general information and clinical details of the pediatric patients, details on concomitant diseases and symptoms, liver function evaluation indicators, laboratory test results, as well as information on preoperative coagulation medications and surgical procedures. The data were collected manually and underwent careful proofreading by a qualified professional to ensure accuracy.

This study was approved by the Ethics Committee of the Children’s Hospital of Fudan University (Ethics Number: No.2023144), and informed consent was waived due to the nature of the study.

In this study, hemorrhage was classified into three categories based on ultrasound findings: *Minor Hemorrhage*: Defined as a small amount of ascites present in the hepatorenal space, pelvic cavity, and right anterior superior hepatic space. *Moderate Hemorrhage*: In addition to the findings of minor hemorrhage, anechoic areas were observed in the gallbladder bed, surrounding the bladder, in the omental sac, and around the spleen. *Massive Hemorrhage*: Characterized by the presence of anechoic areas around the liver and spleen, within the pelvic cavity, and in the intestinal loops, with the mesentery and intestines floating in these anechoic areas.

Due to the limited number of cases categorized as moderate and massive bleeding, a separate classification and statistical analysis for these groups was not feasible. Consequently, all three categories of hemorrhage were combined and analyzed as a single outcome indicator of bleeding in subsequent analyses. Additionally, the term “Needle depth” refers to the length of the puncture needle inserted into the liver tissue after penetrating the pleura.

The literature review indicates that Corticosteroid drugs, Immunosuppressants, Various monoclonal antibodies, and β-lactam antibiotics may affect the coagulation system through different mechanisms (such as influencing platelet function, coagulation factor synthesis, or vascular endothelial integrity). Based on these pharmacological mechanisms, we have included these four classes of drugs in the analysis of factors influencing post-liver biopsy bleeding. Corticosteroid drugs primarily included intravenous and oral preparations: methylprednisolone, dexamethasone, hydrocortisone, and prednisone. Immunosuppressants included: steroids (as mentioned above), cyclophosphamide, mycophenolatemofetil, methotrexate, azathioprine, thiopurine, allopurinol tablets, cyclosporine, tacrolimus, sirolimus. Various monoclonal antibodies included: rituximab, vedolizumab, trastuzumab, alemtuzumab, and cetuximab. Additionally, β-lactam antibiotics included: Penicillins, Cephalosporins, Carbenicillins, Cephamycins, Monocyclic β-lactam antibiotics, Typical β-lactam antibiotics, and β-lactamase inhibitor antibiotics. AAR = AST(U/L)/ALT(U/L); FIB4 = Age(years)*AST(U/L)/PLT(10^9^/L)*ALT^1/2^(U/L); APRI=(AST/ULN)*100/PLT(10^9^/L); GPR=(GGT/ULN) *100/PLT (10^9^/L).

### Statistics analysis

A retrospective analysis was conducted on the preoperative data of 1,665 children who underwent percutaneous liver biopsy. The children were divided into a control group (1,443 cases) and a bleeding group (222 cases) based on the presence or absence of bleeding. Data processing and statistical analysis were performed using SPSS 26.0 and R 4.2.1 software. Continuous variables are presented as median(IQR) while categorical variables are expressed as frequency and percentage. We used t-test to compare continuous variables and the chi-square test to compare categorical variables.

Before analysis, continuous variables were converted to binary form for clinical utility. While this dichotomization has statistical limitations, it provides clear, actionable thresholds that facilitate rapid, practical decision-making in clinical settings. Robert first reported the method of regression shrinkage and selection using the LASSO (Least Absolute Shrinkage and Selection Operator) approach in 1996 [9]. Compared to traditional regression methods, this approach can handle a larger number of potential predictors when many variables are present, allowing for the selection of the variables most relevant to the disease. Therefore, this study aims to utilize Lasso regression analysis to calculate the Lambda value based on cross-validation, with the optimal Lambda value corresponding to the minimum error being used as the criterion for selecting feature variables. The dataset was divided into a training set and a validation set in a 7:3 ratio. Variables with statistically significant differences were included in a binary logistic regression model, and a predictive model was constructed using stepwise bidirectional regression. The nomogram was plotted using the Nomogram function from the RMS(Regression Modeling Strategies) package, and the model was internally validated. Model evaluation was performed using the receiver operating characteristic curve (ROC curve), area under the curve (AUC), calibration plot, and decision curve analysis (DCA).

## Results

### Population characteristics

A total of 1,665 eligible children who underwent percutaneous liver biopsy were included in the study, comprising 1,057 males and 608 females. The age was 2.35[0.79, 7.46] years, and the BMI was 16 [14.58, 17.45]. Among these, there were 136 male and 86 female children in the bleeding group, while the non-bleeding group included 921 male and 522 female children.(Table 1).

**Table 1:** Basic clinical characteristics of enrolled patients

Variables M(Q1, Q3) or *n*(%)	Total (*n* = 1665)	Bleeding (*n* = 222)	Non-bleeding (*n* = 1443)	*P*
Weight(kg)	14.00 (8.50, 25.50)	14.00 (8.50, 25.50)	14.40 (9.00, 25.00)	0.60
Age(years)	3.01(0.78, 7.66)	2.97 (0.77, 7.67)	3.36 (1.06, 7.59)	0.22
BMI	16.40 (15.10, 18.10)	16.50 (15.20, 18.10)	16.00 (15.00, 17.58)	0.04
Needle Depth(mm)	8.90 (7.60, 10.70)	9.00 (7.70, 10.70)	8.50 (7.32, 10.00)	< 0.01
FIB (g/L)	2.46(2.06, 2.93)	2.46 (2.07, 2.92)	2.42 (1.94, 3.01)	0.40
Alb (g/L)	41.00 (37.80, 43.70)	41.10 (38.00, 43.85)	40.10 (36.60, 42.98)	< 0.01
PAB (mg/L)	167.26(116.20, 209.40)	167.26 (119.00, 211.45)	150.65 (100.00, 194.30)	< 0.01
ChE(U/L)	6072.00 (4197.00, 7822.00)	6149.00 (4292.00, 7863.00)	5795.50 (3623.50, 7531.00)	0.05
Glu(mmol/L)	4.34(3.80, 4.82)	4.33 (3.79, 4.79)	4.38 (3.86, 4.89)	0.21
HCT(%)	36.10(32.60, 38.90)	36.20 (32.80, 38.90)	35.60 (31.42, 38.45)	0.07
INR	1.00 (0.94, 1.09)	1.00 (0.94, 1.09)	1.02 (0.95, 1.12)	0.045
APTT(s)	37.10(33.40, 41.60)	36.90 (33.40, 41.30)	38.35 (34.10, 43.80)	< 0.01
PTA(%)	96.00 (84.00, 109.00)	97.00 (85.00, 109.00)	95.00 (80.00, 107.00)	0.04
PT(s)	13.30 (12.60, 14.20)	13.30 (12.60, 14.20)	3.50 (12.70, 14.50)	0.04
TBA(umol/L)	16.60 (5.50, 93.30)	15.50 (5.30, 89.45)	25.95 (7.60, 118.20)	< 0.01
ALP(U/L)	308.00 (231.70, 427.00)	308.00 (234.55, 426.05)	311.50 (212.50, 429.82)	0.15
ALT(U/L)	144.80 (61.30, 317.00)	146.60 (65.00, 321.50)	118.60 (46.52, 287.25)	0.03
AST(U/L)	126.90 (63.00, 291.90)	128.00 (64.00, 287.40)	112.20 (54.33, 295.98)	0.24
DB/TB	0.44 (0.36, 0.73)	0.44 (0.35, 0.72)	0.48 (0.37, 0.76)	0.01
DB(umol/L)	4.50 (2.60, 58.50)	4.30 (2.60, 54.15)	6.55 (2.92, 91.97)	< 0.01
TB(umol/L)	12.10 (7.00, 78.70)	11.80 (6.95, 73.00)	15.25 (7.20, 120.55)	0.02
GGT(U/L)	58.40 (26.00, 129.00)	57.00 (25.25, 122.80)	73.45 (34.92, 178.80)	< 0.01
HB (g/L)	120.00 (107.00, 130.00)	120.00 (108.00, 130.00)	118.00 (105.00, 129.00)	0.09
PLT(*10^9^/L)	300.00 (224.00, 382.00)	307.00 (235.00, 388.00)	247.50 (154.75, 335.50)	< 0.01
AAR	1.00 (0.68, 1.51)	0.99 (0.67, 1.50)	1.16 (0.75, 1.63)	0.01
FIB4	0.02 (0.00, 0.05)	0.02 (0.00, 0.05)	0.02 (0.01, 0.08)	< 0.01
APRI	0.01 (0.00, 0.02)	0.01(0.00, 0.02)	0.01 (0.01, 0.02)	< 0.01
GPR	0.01 (0.00, 0.02)	0.00(0.00, 0.01)	0.01 (0.00, 0.03)	< 0.01
Gender				0.46
Female	608 (36.5)	86 (38.7)	522 (36.2)	
Male	1057 (63.5)	136 (61.3)	921 (63.8)	
Pre K1				< 0.01
No	893 (53.6)	99 (44.6)	794 (55.0)	
Yes	772 (46.4)	123 (55.4)	649 (45.0)	
Needle position				0.39
Intercostal	1368 (82.2)	187 (84.2)	1181 (81.8)	
Out of intercostal	297 (17.8)	35 (15.8)	262 (18.2)	
Anemia				0.046
No	1287 (77.3)	160 (72.1)	1127 (78.1)	
Yes	378 (22.7)	62 (27.9)	316 (21.9)	
Splenomegaly				< 0.001
No	1178 (70.8)	129 (58.1)	1049 (72.7)	
Yes	487 (29.3)	93 (41.9)	394 (27.3)	
Cholestasis				0.06
No	1191 (71.5)	147 (66.2)	1044 (72.4)	
Yes	474 (28.5)	75 (33.8)	399 (27.6)	
Pre-β-lactams				0.02
No	1436 (86.3)	180 (81.1)	1256 (87.0)	
Yes	229 (13.8)	42 (18.9)	187 (13.0)	
Pre-ISA				0.03
No	1493 (89.7)	190 (85.6)	1303 (90.3)	
Yes	172 (10.3)	32 (14.4)	140 (9.7)	
Post Liver Transplantation				0.20
No	1602 (96.2)	217 (97.8)	1385 (96.0)	
Yes	63 (3.8)	5 (2.3)	58 (4.0)	
Pre-Corticosteroid				0.01
No	1510 (90.7)	191 (86.0)	1319 (91.4)	
Yes	155 (9.3)	31 (14.0)	124 (8.6)	
AIH				0.62
No	1607 (96.5)	213 (96.0)	1394 (96.6)	
Yes	58 (3.5)	9 (4.1)	49 (3.4)	
EBV				0.40
No	1586 (95.3)	209 (94.1)	1377 (95.4)	
Yes	79 (4.7)	13 (5.9)	66 (4.6)	
CMV				0.81
No	1608 (96.6)	215 (96.9)	1393 (96.5)	
Yes	57 (3.4)	7 (3.2)	50 (3.5)	
Fibrosis liver				0.17
No	1538 (92.4)	200 (90.1)	1338 (92.7)	
Yes	127 (7.6)	22 (9.9)	105 (7.3)	

*AAR* AST/ALT ratio, *AIH* Autoimmune hepatitis, *Alb* Albumin, *ALP* Alkaline phosphatase, *ALT* Alanine transferase, *APRI* Aspartate aminotransferase to platelet ratio index, *APTT* Activated partial thromboplastin time, *AST* Aspartate transaminase, *BMI* Body mass index , *ChE* Cholinesterase, *CMV* Cytomegalovirus, *DB* Direct bilirubin, *EBV* Epstein-barr virus, *FIB* Fibrinogen, *FIB4* Fibrosis index based on the four factors, *GGT* Gamma-glutamyl-transferase, *Glu* glucose, *GPR* Gamma-glutamyl transferase to platelet ratio, *HB* Hemoglobin, *HCT* Hematocrit, *INR* International normalized ratio, *PAB* Prealbumin, *PLT* Platelets

*Pre K1* K1 were administered within 24 h before liver biopsy, *Pre-Corticosteroid* Corticosteroid were administered within 24 h before liver biopsy, *AIH* Autoimmune Hepatitis, *Pre-ISA* Immunosuppressive agents were administered within 24 h before liver biops, Pre-β-lactams, β-lactam antibiotic were administered within 24 h before liver biops, *PT*, Prothrombin time, *PTA* Prothrombin time activity, *TB* Total bilirubin, *TBA* Total bile acids

The assignment rules for categorical variables are defined as follows: for bleeding, 0 represents the non-bleeding group and 1 represents the bleeding group; for gender, 1 indicates male and 2 indicates female; for all other variables, including Pre K1, Anaemia, Splenomegaly, Pre-Corticosteroid, Pre-β-lactams, Pre-ISA, Post Liver Transplantation, AIH, EBV, CMV, and Fibrosis liver, 1 denotes “No” and 2 denotes “Yes”. For clinical convenience, the ROC curve was used to calculate the cutoff values, sensitivity, and specificity corresponding to the maximum Youden index for each continuous variable. Additionally, based on the cutoff values, the continuous variables were converted into binary variable values, as detailed in Table 2.

**Table 2:** Cutoff values for continuous variables

Variables	Cut-off value	Specificity	Sensitivity
HCT (%)	33.35	0.721	0.355
BMI	16.85	0.441	0.664
GGT(U/L)	121.1	0.749	0.383
PLT (*10^9^/L)	203.5	0.843	0.388
PT(s)	15.05	0.883	0.206
Needle Depth(mm)	8.25	0.634	0.467
ALT(U/L)	63.35	0.756	0.360
FIB4	0.065	0.82	0.32
APRI	0.012	0.66	0.45
GPR	0.02	0.81	0.39

Assignment of values: Continuous variables were classified as 1 if they were less than or equal to the cutoff value, and as 2 if they were greater than the cutoff value

### Identifying predictors of bleeding by LASSO regression

Due to the correlation among different independent variables, we performed LASSO regression analysis on all independent variables to identify the most representative predictors of postoperative hemorrhage. A 10-fold cross-validation was conducted to select the lambda parameter, and a dashed line was drawn at the optimal parameter. The best log(λ) selected in the figure is 0.00562077, at which point the fitted LASSO regression model is deemed the most appropriate.(See Supplement Fig. 1ab) The intersecting 16 independent variables are the selected non-zero coefficient variables, including the results from the LASSO analysis: Pre K1、Splenomegaly 、Pre-β-lactams 、Post Liver Transplantation、Pre-Corticosteroid、BMI、Needle Depth、HCT、PT、ALT、GGT、PLT、FIB4、Fibrosis liver、APRI、GPR.

### Training and validation cohort

The total dataset was divided into a training set and a validation set in a 7:3 ratio. A comparison was made between the bleeding group and the control group for the divided datasets. (See Supplement Table 1)

### Univariate and multivariate logistic regression analysis of selected predictors in training cohort

The significant variables identified by Lasso regression were incorporated into the multivariate logistic regression analysis.(Table 3) Through stepwise bidirectional regression, the following parameters were significantly different between the bleeding and non-bleeding groups in the training cohort: Pre-Corticosteroid, Post Liver Transplantation, Needle Depth>8.25 mm, ALT>63.35U/L, PT>15.05s, PLT>203.5*10^9^/L and GPR>0.02.

**Table 3:** Univariate and multivariate logistic regression analysis of factors influencing bleeding in liver biopsy children

Variables	One-way logistic regression analysis					Multivariable logistic regression analysis				
	β	S.E	Z	*P*	OR (95%CI)	β	S.E	Z	*P*	OR (95%CI)
Pre K1										
No					1.00 (Reference)					
Yes	0.46	0.18	2.59	**0.010**	1.59 (1.12 ~ 2.26)					
Splenomegaly										
No					1.00 (Reference)					
Yes	0.68	0.18	3.78	**< 0.001**	1.98 (1.39 ~ 2.82)					
Pre-β-lactams										
No					1.00 (Reference)					
Yes	0.52	0.22	2.34	**0.019**	1.68 (1.09 ~ 2.60)					
Post Liver Transplantation										
No					1.00 (Reference)					1.00 (Reference)
Yes	−0.78	0.60	−1.30	0.195	0.46 (0.14 ~ 1.49)	−1.74	0.63	2.75	**0.006**	0.18 (0.05 ~ 0.61)
Pre-Corticosteroid										
No					1.00 (Reference)					1.00 (Reference)
Yes	0.82	0.26	3.21	**0.001**	2.28 (1.38 ~ 3.76)	0.69	0.29	2.42	**0.016**	2.00 (1.14 ~ 3.51)
BMI										
≤ 16.85					1.00 (Reference)					
>16.85	−0.40	0.18	−2.16	**0.031**	0.67 (0.47 ~ 0.96)					
Needle Depth										
≤ 8.25					1.00 (Reference)					1.00 (Reference)
>8.25	−0.57	0.18	−3.09	**0.002**	0.57 (0.39 ~ 0.81)	−0.71	0.20	−3.63	**< 0.001**	0.49 (0.34 ~ 0.72)
ALT										
≤ 63.35					1.00 (Reference)					1.00 (Reference)
>63.35	−0.45	0.18	−2.43	**0.015**	0.64 (0.45 ~ 0.92)	−0.47	0.20	−2.33	**0.020**	0.63 (0.42 ~ 0.93)
GGT										
≤ 121.1					1.00 (Reference)					
>121.1	0.59	0.19	3.17	**0.001**	1.81 (1.26 ~ 2.62)					
HCT										
≤ 33.35					1.00 (Reference)					
>33.35	−0.38	0.18	−2.05	**0.040**	0.69 (0.48 ~ 0.98)					
PT										
≤ 15.05					1.00 (Reference)					1.00 (Reference)
>15.05	0.82	0.22	3.72	**< 0.001**	2.27 (1.47 ~ 3.51)	0.56	0.24	2.36	**0.019**	1.75 (1.10 ~ 2.78)
PLT										
≤ 203.5					1.00 (Reference)					1.00 (Reference)
>203.5	−1.20	0.19	−6.27	**< 0.001**	0.30 (0.21 ~ 0.44)	−0.95	0.21	−4.46	**< 0.001**	0.39 (0.25 ~ 0.59)
Fibrosis liver										
No					1.00 (Reference)					
Yes	0.46	0.28	1.62	0.106	1.58 (0.91 ~ 2.76)					
APRI										
≤ 0.012					1.00 (Reference)					
>0.012	0.44	0.18	2.47	**0.013**	1.56 (1.10 ~ 2.21)					
GPR										
≤ 0.02					1.00 (Reference)					1.00 (Reference)
>0.02	0.97	0.19	5.12	**< 0.001**	2.64 (1.82 ~ 3.84)	0.79	0.21	3.72	**< 0.001**	2.20 (1.45 ~ 3.33)
FIB4										
≤ 0.065					1.00 (Reference)					
>0.065	0.58	0.20	2.95	**0.003**	1.79 (1.22 ~ 2.65)					

*OR* Odds Ratio, *CI* Confidence Interval

### Construction of the nomogram

**Fig. 1:**
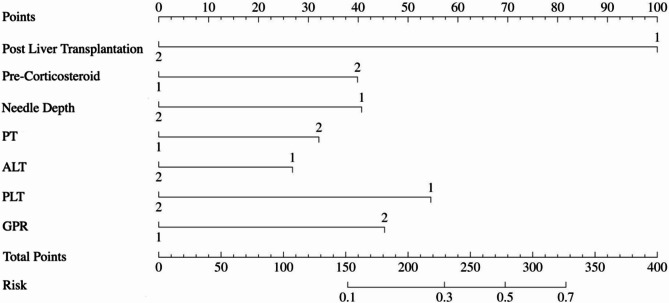
Nomogram for predicting bleeding in pediatric percutaneous liver biopsy

Based on the final independent influencing factors, a predictive nomogram was generated. (Fig. 1)Details of the characteristics of the patients with severe bleeding are provided in Table 4. The detailed distribution of the 7 predictors included in the final model across the bleeding and non-bleeding groups is presented in Supplement Table 2.

**Table 4:** General characteristics of patients with severe bleeding

No.	Gender	Age (years)	Weight	Diagnosis	Post Liver Transplantation	Pre- Corticosteroid	Needle Depth	PT	ALT	PLT	GPR	Treatment	Outcome
1	Male	7.6	23.5	Liver dysfunction	+	—	—	—	—	+	+	Volume expansion, pRBC and plasma transfusion	Recovered
2	Male	7.7	30	Cholestasis	+	—	—	—	—	+	+	Volume expansion, pRBC and platelet transfusion	Recovered
3	Male	8.0	29.8	Severe hepatitis	+	—	—	—	—	+	+	Volume expansion, pRBC and plasma transfusion, transfer to ICU.	Recovered
4	Male	7.3	19.5	Chronic hepatitis B	+	—	—	—	—	—	+	Volume expansion, pRBC and plasma transfusion	Recovered
5	Female	2.8	11.9	Liver dysfunction	+	—	+	—	+	+	+	Volume expansion, pRBC and plasma transfusion, transfer to ICU.	Recovered
6	Male	14.9	48	Liver dysfunction after liver transplantation	—	—	—	—	—	+	+	Volume expansion, plasma transfusion	Recovered

“+” indicates that the risk factor is scored in the nomogram, and “—” indicates otherwise

### Internal validation

**Fig. 2:**
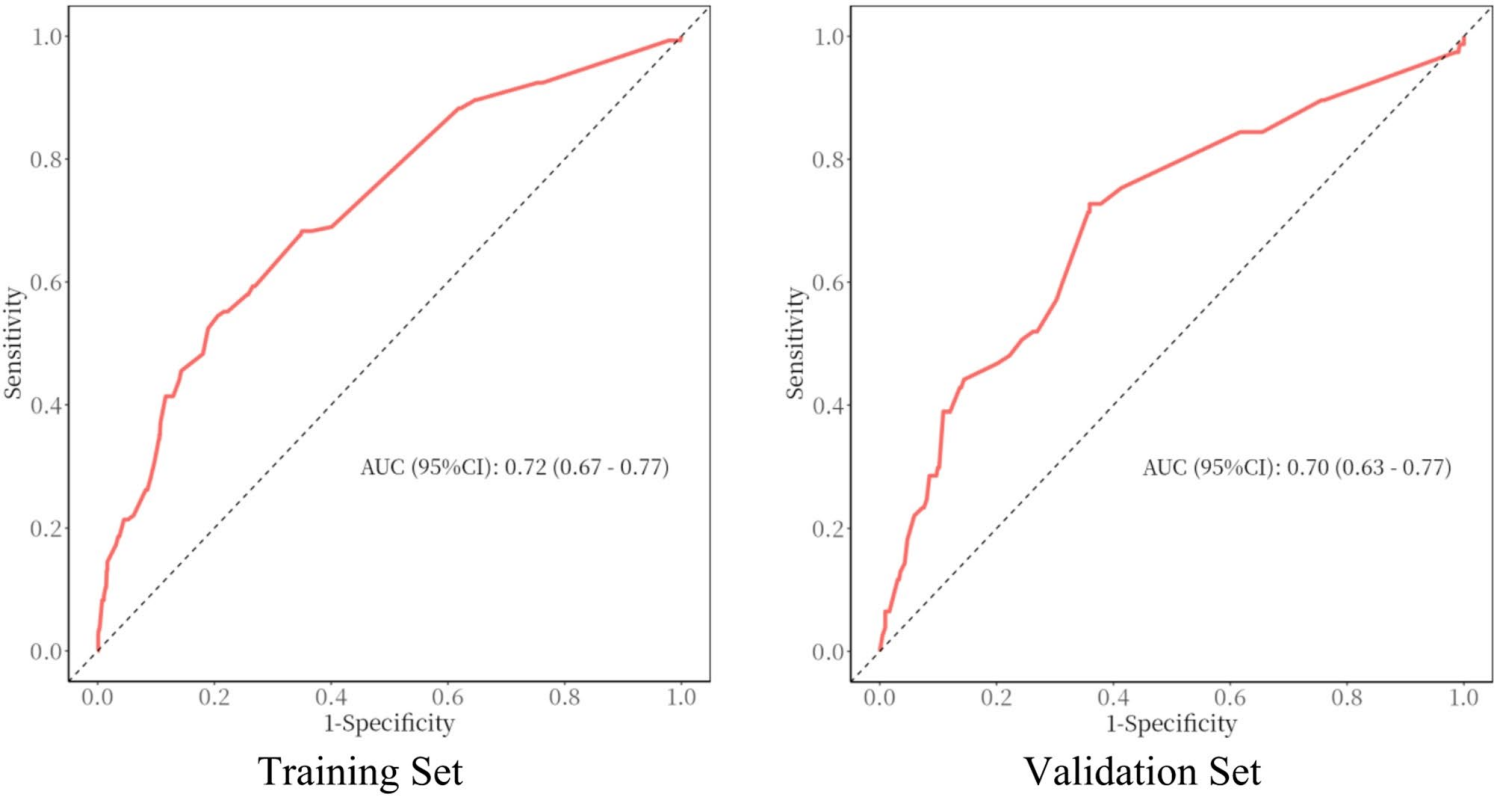
ROC Curves for the Training Set and Validation Set

Discrimination is assessed using the ROC curve and the AUC. The critical value (cut-off value) of the ROC curve is calculated when the Youden index is maximized, along with the corresponding sensitivity and specificity. We used Swets’s criteria, which categorize values as follows: 0.5–0.6 (bad), 0.6–0.7 (poor), 0.7–0.8 (satisfactory), 0.8–0.9 (good), and 0.9–1.0.9.0 (excellent) [10]. The AUC for the training set was 0.720 (95% CI: 0.675–0.765), and the AUC for the validation set was 0.700 (95% CI: 0.633–0.767). (Fig. 2)

**Fig. 3:**
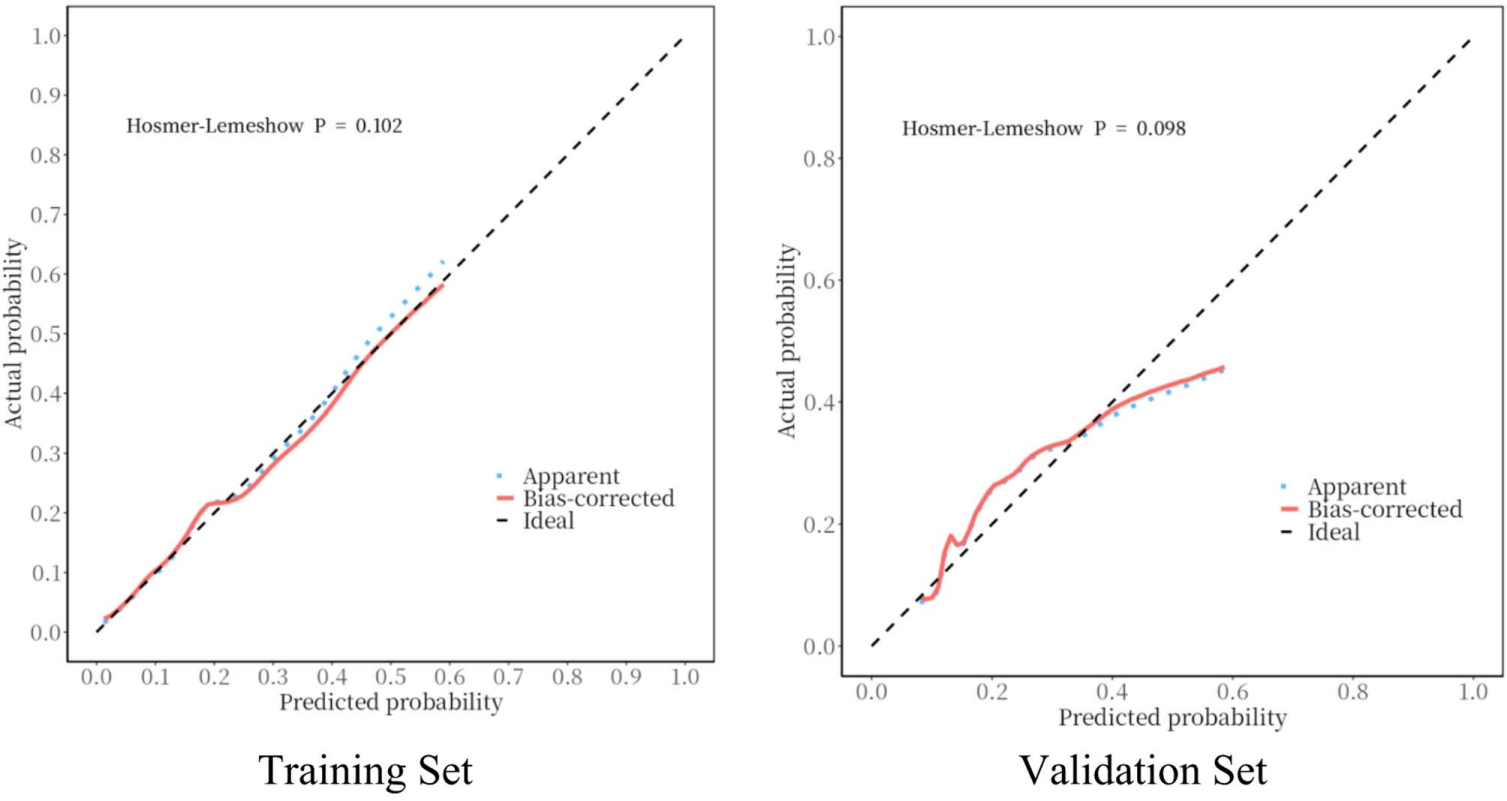
Calibration Curves for the Training Set and Validation Set

Model calibration was evaluated using calibration curves and Hosmer-Lemeshow tests on both training and validation sets (Fig. 3). For the training set, the test yielded *p* = 0.102; for the validation set, *p* = 0.098. Both *p*>0.05, indicating acceptable overall calibration for the model on both sets. In Fig. 3, the bias-corrected curve of the training set aligned well with the ideal line across the 0.0–0.6 predicted probability range. The validation set’s curve fitted well at 0.1–0.4 but slightly underestimated the actual bleeding risk when the predicted probability > 0.4. The model meets the basic clinical reliability requirements; however, high-risk predictions should be combined with other clinical information for informed judgment.

**Fig. 4:**
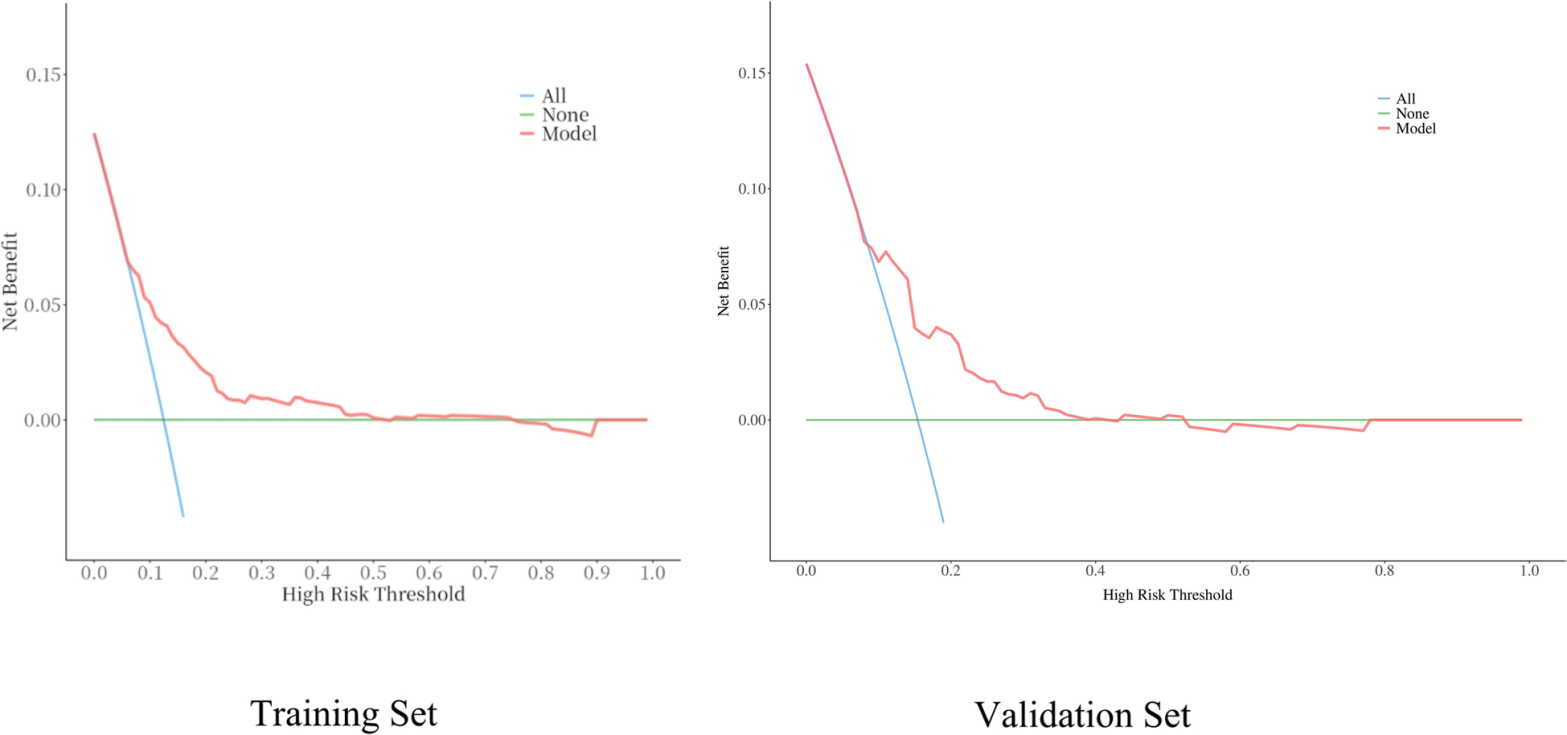
Decision Curve Analysis for the Training Set and Validation Set

Clinical effectiveness was assessed using DCA. The further the DCA curve is from the x-axis and y-axis, the greater the benefit to patients, indicating better clinical effectiveness of the model. (Fig. 4)

## Discussion

Most percutaneous liver biopsy procedures are considered safe, with a low incidence of postoperative complications. However, as an invasive procedure, there are inherent risks associated with percutaneous liver biopsy. Potential complications may include pain, bleeding, biliary peritonitis, arteriovenous fistula, penetration of abdominal viscera, vasovagal reactions, pneumothorax, and hemothorax. Among these, bleeding is regarded as the most severe complication and can result in mortality in critical cases.

In this study, the rate of bleeding was found to be 13.3%, with the majority of cases involving minor bleeding that was effectively managed without leading to significant complications. This finding aligns with previously reported incidence rates, which range from 4.6% to 18%. This study preliminarily establisheda predictive model for the risk of bleeding associated with percutaneous liver biopsy in children, comprising seven variables: Post Liver Transplantation, Pre-Corticosteroid, Needle Depth, PT, ALT, PLT, and GPR.

Among the variables analyzed, post-liver transplantation showed an OR of 0.18 for bleeding risk in children, suggesting a potential protective effect. However, this finding is based on a small subgroup—only 5 cases (2.3%) in the bleeding group and 58 cases (4.0%) in the non-bleeding group—resulting in a wide 95% confidence interval (0.05–0.61) and limited stability. Thus, this result should be interpreted cautiously as exploratory rather than definitive. Previous studies [11] propose that liver transplantation may reduce bleeding risk through mechanisms such as hypercoagulability during the perioperative period and up to one year post-transplantation, driven by immunosuppressants. These medications activate endothelial cells, releasing von willebrand factor (VWF), coagulation factor VIII (FVIII), tissue factor pathway inhibitor (TFPI), and plasminogen activator inhibitor-1 (PAI-1) [12]– [13], while also inhibiting fibrinolysis [14]. Additionally, the transplanted liver may offer a better coagulation status than the diseased liver [15]. At our center, all pediatric transplant biopsies are performed by experienced attending physicians, which may further reduce complications [16–18]. However, given the small sample size and the natural scarcity of post-transplantation cases, these findings should be considered in the context of clinical practice. We present this hypothesis as a directional clue for future research, but emphasize that the results are not stable enough to inform clinical decisions. Larger studies are needed to validate these observations and provide more robust evidence.

Pre-Corticosteroid refers to the administration of corticosteroids within 24 h of surgery. The risk of bleeding in children receiving corticosteroids is 2.00 times greater than in those not receiving these medications, indicating that corticosteroid use is associated with an increased risk of bleeding. This finding is consistent with previous studies [19]. Corticosteroids are primarily used in the treatment of severe liver diseases in children, including autoimmune hepatitis [20], Epstein-Barr virus infections [21], and pediatric population-specific drug-induced liver injury [22], characterized by elevated bilirubin levels and severe hepatic dysfunction. For instance, the standard treatment regimen for children with autoimmune hepatitis [23] involves the administration of high initial doses of corticosteroids, which are gradually tapered following the introduction of azathioprine. Studies have shown that corticosteroids influence thromboxane formation by inhibiting the release of arachidonic acid from membrane phosphatidylinositol and phosphatidylcholine, which can result in increased vascular fragility. This mechanism may lead to the inhibition of vascular contraction and platelet activation, thereby contributing to a higher incidence of bleeding [24]– [25].

Needle Depth indicates that a depth greater than 8.25 mm carries a bleeding risk that is 0.49 times compared to a depth of 8.25 mm or less, suggesting that a needle depth exceeding 8.25 mm is associated with a decreased risk of bleeding. This finding aligns with existing research; for instance, Cao et al. [26] analyzed the risk factors for bleeding complications during percutaneous liver biopsy guided by ultrasound or CT in patients with space-occupying liver lesions, and found that needle depth is a significant risk factor for bleeding. Although studies specifically addressing liver biopsy depth are limited, some scholars have developed precise calculations for renal biopsy needle depth based on patient height and weight [27]. Their findings indicated that this method significantly reduced the incidence of bleeding-related complications following renal biopsies. Given that the kidneys are also solid organs and undergo percutaneous procedures under ultrasound guidance, these insights may have relevant implications for the depth of liver biopsy needles.

PT exceeding 15.05 s is associated with a bleeding risk that is 1.75 times greater than that of patients with a PT of 15.05 s or less, suggesting that a PT greater than 15.05 s correlates with an elevated risk of hemorrhage. In individuals with liver disease, hepatocellular injury leads to abnormalities and reductions in the synthesis of clotting factors, which adversely affect hemostatic function. PT is frequently employed as a parameter for assessing coagulation status in patients with compromised liver function and for estimating the bleeding risk in this population. In accordance with prior studies, prolonged PT is identified as a significant risk factor for bleeding complications during percutaneous liver biopsy. Research has demonstrated that an increase in PT of more than 3 s (14.15 ± 2.34 vs. 13.61 ± 1.63) markedly raises the incidence of bleeding (10.5% vs. 7%) [18]. Additionally, some studies have indicated that while PT is correlated with a higher incidence of bleeding, it does not serve as a predictive factor for hemorrhagic events [4].

ALT levels greater than 63.35 U/L are associated with a bleeding risk that is 0.63 times that of patients with ALT levels of 63.35 U/L or lower, indicating that ALT levels ≤ 63.35 U/L are linked to an increased risk of hemorrhage. ALT is predominantly utilized as a biomarker for liver inflammation or damage. Chronic liver disease represents a severe and advanced hepatic condition, encompassing a range of disorders such as viral hepatitis, cirrhosis, hepatic failure, and non-alcoholic fatty liver disease [28]. Due to the nature of chronic disease, ALT levels may not be markedly elevated and are often found to be below normal limits [29]. In pediatric patients with chronic liver disease, the progression of the condition leads to substantial parenchymal damage, persistent inflammatory responses, and ongoing activation of wound healing mechanisms. This is predominantly characterized by the excessive accumulation of extracellular matrix components, which is maintained by estrogen-responsive hepatic myofibroblasts. Consequently, these patients exhibit a greater degree of hepatic fibrosis and sustained pathological angiogenesis [30], which complicates the procedural challenges associated with liver puncture and increases the likelihood of bleeding.

GPR greater than 0.02 is associated with a 2.20-fold increased risk of bleeding compared to GPR values of 0.02 or lower, indicating that a GPR >0.02 is linked to a heightened risk of hemorrhage. GPR is a novel biomarker developed by Lemoine et al. [31] for predicting hepatic fibrosis and cirrhosis in patients with chronic hepatitis B virus infection. The predictive efficacy of this indicator for assessing hepatic fibrosis has been validated by multiple studies [32–34]. Additionally, investigations suggest that GPR can serve as an independent predictor of adverse outcomes related to hepatic fibrosis and hepatocellular carcinoma [35]. From a pathological perspective, the liver, as the central organ for synthesizing coagulation factors and clearing fibrinolytic substances, directly influences the balance of hemostasis and coagulation systems. Liver fibrosis, a critical stage in the progression of chronic liver disease, is a dynamic and continuous process: as fibrosis worsens, hepatocytes gradually become damaged and lose function. On one hand, this leads to impaired platelet production and regulation (studies have confirmed a significant negative correlation between platelet count and the severity of liver fibrosis, meaning that the more severe the fibrosis, the more pronounced the thrombocytopenia). On the other hand, it causes primary hemostatic dysfunction, coagulation disorders, and fibrinolytic system imbalance. These pathological changes collectively increase the risk of bleeding complications. If liver fibrosis progresses further, it may develop into cirrhosis or even hepatocellular carcinoma. Both liver fibrosis and cirrhosis are closely associated with the risk of bleeding during liver biopsy—studies have clearly indicated that cirrhosis itself is a significant risk factor for bleeding complications related to liver biopsy [26]. This further suggests that the degree of liver fibrosis (and its progression to cirrhosis) may be an important potential indicator for assessing the risk of bleeding during liver biopsy. It also explains, at a mechanistic level, why biomarkers related to liver fibrosis, such as GPR, are associated with bleeding risk.

PLT greater than 203.5*10^9^/L is associated with a 0.39-fold decreased risk of bleeding compared to a PLT of 203.5*10^9^/L or lower, indicating that PLT ≤ 203.5*10^9^/L is linked to an increased risk of hemorrhage, which is consistent with previous research findings. PLT is a critical indicator of coagulation function. A study by Steeff et al. [36] demonstrated that a PLT < 60 *10^9^/L (6000/mm³) is associated with a higher likelihood of puncture-related complications. However, our study presents more conservative data, which may be attributed to the specific age of the pediatric population, leading to more cautious clinical practices during procedures. Existing research [37] shows that low platelet counts significantly impact bleeding rates, and it is generally not recommended to perform liver biopsies on patients with low PLT values. Consequently, the PLT values of pediatric patients undergoing liver biopsies at our institution are predominantly maintained within the normal range of 100–300*10^9^/L.

This study has several important limitations that need to be acknowledged. First, the retrospective, single-center design may introduce bias and limit the generalizability of the findings. Although we ensured data completeness, retrospective studies are prone to missing or inconsistent records. Second, unmeasured confounders, such as coagulation profiles, liver pathology, or operator technique, may have influenced bleeding risk but were not fully accounted for in the analysis. These factors likely contribute to the model’s moderate performance. Third, due to the limited number of moderate and massive bleeding cases, we combined all bleeding categories into a composite endpoint. While this improvement in statistical power may be beneficial, it may also obscure specific predictors of clinically significant massive bleeding, thereby limiting the model’s ability to identify patients at high risk.

The model’s discriminative ability is only moderate, representing a preliminary framework that requires further optimization and external validation. It is important to avoid overinterpreting the model’s predictive capabilities. Future studies should focus on larger, multi-center, prospective designs to validate and refine the model, incorporating additional variables and advanced modeling techniques to enhance predictive accuracy. In our subsequent work, we will focus specifically on predicting massive bleeding through dedicated research, providing more targeted risk prevention and control tools for clinical practice, thereby enhancing the practical application value of our findings. We believe that these efforts will further optimize the predictive capabilities of our model and increase its value in clinical practice.

## Conclusion

This study retrospectively analyzed data from pediatric patients undergoing percutaneous liver biopsy to develop and internally validate a bleeding risk prediction model. The model demonstrated moderate discriminative ability, indicating its potential to predict post-biopsy bleeding risk to some extent. However, predictive accuracy requires further improvement. As a preliminary tool, the model needs additional optimization and external validation before its clinical utility can be fully assessed. Future multi-center studies with larger datasets are essential to refine the model and enhance its ability to identify and manage high-risk patients effectively, ultimately reducing bleeding complications in children.

## Supplementary Information

Supplementary Material 1.

## Data Availability

The datasets generated and analysed during the current study are not publicly available due to ethical restrictions on sharing of de-identified data, but are available from the corresponding author on reasonable request.

## Abbreviations

AAR: AST/ALT ratio
AIH: Autoimmune hepatitis
Alb: Albumin
ALP: Alkaline phosphatase
ALT: Alanine transferase
APRI: Aspartate aminotransferase to platelet ratio index
APTT: Activated partial thromboplastin time
AST: Aspartate transaminase
AUC: Area under the curve
BMI: Body mass index
ChE: Cholinesterase
CMV: Cytomegalovirus
DB: Direct bilirubin
DCA: Decision curve analysis
EBV: Epstein-barr virus
FIB: Fibrinogen
FIB4: Fibrosis index based on the four factors
FVIII: Coagulation factor VIII
GGT: Gamma-glutamyl-transferase
Glu: Glucose
GPR: Gamma-glutamyl transferase to platelet ratio
HB: Hemoglobin
HCT: Hematocrit
INR: International normalized ratio
LASSO: Least absolute shrinkage and selection operator
PAB: Prealbumin
PAI-1: Plasminogen activator inhibitor-1
PLT: Platelets
Pre K1: K1 were administered within 24 hours before liver biopsy
Pre-Corticosteroid: Corticosteroid were administered within 24 hours before liver biopsy
AIH: Autoimmune Hepatitis
Pre-ISA: Immunosuppressive agents were administered within 24 hours before liver biopsy
Pre-β-lactams: β-lactam antibiotic were administered within 24 hours before liver biopsy
PT: Prothrombin time
PTA: Prothrombin time activity
RMS: Regression modeling strategies
ROC curve: Receiver operating characteristic curve
TB: Total bilirubin
TBA: Total bile acids
TFPI: Tissue factor pathway inhibitor
ULN: Upper limit of normal
VWF: Von willebrand factor
