# Supplementary material for: Development and validation of bleeding prediction model for percutaneous liver biopsy in children
Source: BMC Pediatr. 2025 Dec 8;26:187. doi: 10.1186/s12887-025-06341-w (PMC12961818; doi:10.1186/s12887-025-06341-w)
Supplement: Supplementary file 1 — Supplementary Material 1. [file 12887_2025_6341_MOESM1_ESM.docx]

**Supplement**

| 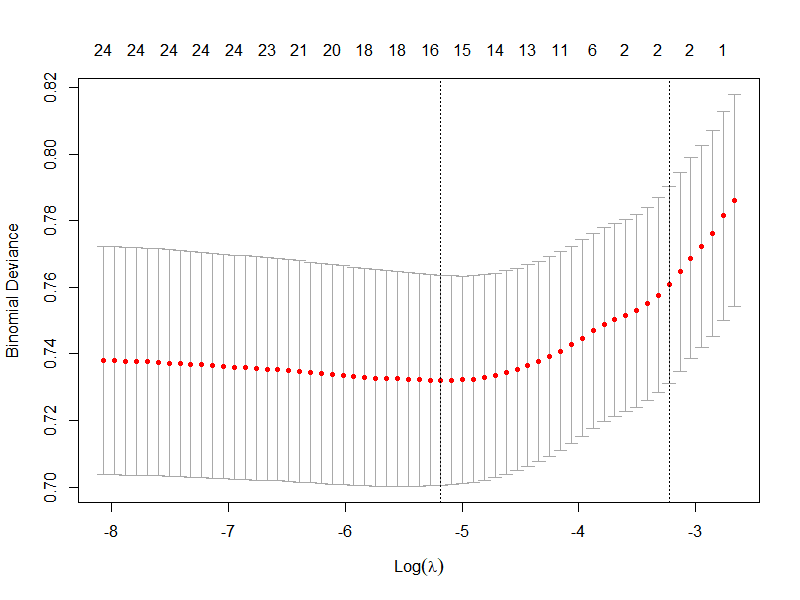 | 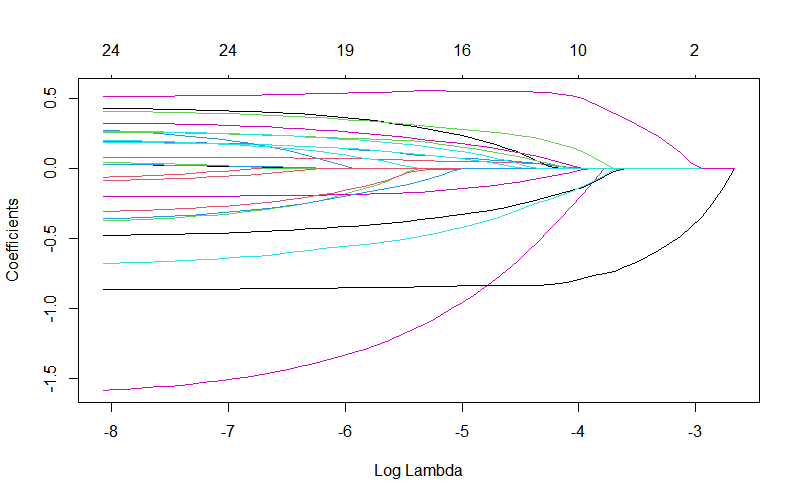 |
| --- | --- |
| **Figure 1(a) Selection of the optimal parameter lambda in the LASSO model** | **Figure 1 (b) Distribution of LASSO coefficients for the 16 variables** |

**Table 1. Chi-square test between the training set and the validation set.**

| Variable | Total (N= 1665) | Train (N= 1165) | Valid (N= 500) | Statistic | P |
| --- | --- | --- | --- | --- | --- |
| Bleeding, N (%) |  |  |  | χ²=2.641 | 0.104 |
| 0 | 1443 (86.67) | 1020 (87.55) | 423 (84.60) |  |  |
| 1 | 222 (13.33) | 145 (12.45) | 77 (15.40) |  |  |
| Gender, N (%) |  |  |  | χ²=0.259 | 0.611 |
| 1 | 1057 (63.48) | 735 (63.09) | 322 (64.40) |  |  |
| 2 | 608 (36.52) | 430 (36.91) | 178 (35.60) |  |  |
| Pre-K1, N (%) |  |  |  | χ²=0.897 | 0.344 |
| 1 | 893 (53.63) | 616 (52.88) | 277 (55.40) |  |  |
| 2 | 772 (46.37) | 549 (47.12) | 223 (44.60) |  |  |
| Anaemia, N (%) |  |  |  | χ²=0.691 | 0.406 |
| 1 | 1287 (77.3) | 894 (76.74) | 393 (78.60) |  |  |
| 2 | 378 (22.7) | 271 (23.26) | 107 (21.40) |  |  |
| Splenomegaly, N (%) |  |  |  | χ²=6.235 | 0.013 |
| 1 | 1178 (70.75) | 803 (68.93) | 375 (75.00) |  |  |
| 2 | 487 (29.25) | 362 (31.07) | 125 (25.00) |  |  |
| Pre-β-lactams , N (%) |  |  |  | χ²=3.929 | 0.047 |
| 1 | 1436 (86.25) | 992 (85.15) | 444 (88.80) |  |  |
| 2 | 229 (13.75) | 173 (14.85) | 56 (11.20) |  |  |
| Pre-ISA, N (%) |  |  |  | χ²=1.244 | 0.265 |
| 1 | 1493 (89.67) | 1051 (90.21) | 442 (88.40) |  |  |
| 2 | 172 (10.33) | 114 (9.79) | 58 (11.60) |  |  |
| Post liver transplantation, N (%) |  |  |  | χ²=1.206 | 0.272 |
| 1 | 1602 (96.22) | 1117 (95.88) | 485 (97.00) |  |  |
| 2 | 63 (3.78) | 48 (4.12) | 15 (3.00) |  |  |
| Pre-Corticosteroid, N (%) |  |  |  | χ²=1.881 | 0.170 |
| 1 | 1510 (90.69) | 1064 (91.33) | 446 (89.20) |  |  |
| 2 | 155 (9.31) | 101 (8.67) | 54 (10.80) |  |  |
| AIH, N (%) |  |  |  | χ²=4.677 | 0.031 |
| 1 | 1607 (96.52) | 1117 (95.88) | 490 (98.00) |  |  |
| 2 | 58 (3.48) | 48 (4.12) | 10 (2.00) |  |  |
| EBV, N (%) |  |  |  | χ²=0.328 | 0.567 |
| 1 | 1586 (95.26) | 1112 (95.45) | 474 (94.80) |  |  |
| 2 | 79 (4.74) | 53 (4.55) | 26 (5.20) |  |  |
| CMV, N (%) |  |  |  | χ²=0.840 | 0.359 |
| 1 | 1608 (96.58) | 1122 (96.31) | 486 (97.20) |  |  |
| 2 | 57 (3.42) | 43 (3.69) | 14 (2.80) |  |  |
| Age, N (%) |  |  |  | χ²=3.159 | 0.076 |
| 1 | 623 (37.42) | 452 (38.80) | 171 (34.20) |  |  |
| 2 | 1042 (62.58) | 713 (61.20) | 329 (65.80) |  |  |
| BMI, N (%) |  |  |  | χ²=1.392 | 0.238 |
| 1 | 956 (57.42) | 658 (56.48) | 298 (59.60) |  |  |
| 2 | 709 (42.58) | 507 (43.52) | 202 (40.40) |  |  |
| NeedleDeepth, N (%) |  |  |  | χ²=1.111 | 0.292 |
| 1 | 852 (51.17) | 606 (52.02) | 246 (49.20) |  |  |
| 2 | 813 (48.83) | 559 (47.98) | 254 (50.80) |  |  |
| HCT, N (%) |  |  |  | χ²=4.520 | 0.034 |
| 1 | 483 (29.01) | 356 (30.56) | 127 (25.40) |  |  |
| 2 | 1182 (70.99) | 809 (69.44) | 373 (74.60) |  |  |
| PT, N (%) |  |  |  | χ²=0.002 | 0.966 |
| 1 | 1451 (87.15) | 1015 (87.12) | 436 (87.20) |  |  |
| 2 | 214 (12.85) | 150 (12.88) | 64 (12.80) |  |  |
| TBA, N (%) |  |  |  | χ²=0.656 | 0.418 |
| 1 | 784 (47.09) | 541 (46.44) | 243 (48.60) |  |  |
| 2 | 881 (52.91) | 624 (53.56) | 257 (51.40) |  |  |
| ALT, N (%) |  |  |  | χ²=2.379 | 0.123 |
| 1 | 490 (29.43) | 356 (30.56) | 134 (26.80) |  |  |
| 2 | 1175 (70.57) | 809 (69.44) | 366 (73.20) |  |  |
| GGT, N (%) |  |  |  | χ²=3.648 | 0.056 |
| 1 | 1215 (72.97) | 866 (74.33) | 349 (69.80) |  |  |
| 2 | 450 (27.03) | 299 (25.67) | 151 (30.20) |  |  |
| PLT, N (%) |  |  |  | χ²=0.009 | 0.923 |
| 1 | 314 (18.86) | 219 (18.80) | 95 (19.00) |  |  |
| 2 | 1351 (81.14) | 946 (81.20) | 405 (81.00) |  |  |
| AAR, N (%) |  |  |  | χ²=0.920 | 0.337 |
| 1 | 993 (59.64) | 686 (58.88) | 307 (61.40) |  |  |
| 2 | 672 (40.36) | 479 (41.12) | 193 (38.60) |  |  |
| FIB4, N (%) |  |  |  | χ²=0.120 | 0.729 |
| 1 | 1330 (79.88) | 928 (79.66) | 402 (80.40) |  |  |
| 2 | 335 (20.12) | 237 (20.34) | 98 (19.60) |  |  |
| Fibrosis liver, N (%) |  |  |  | χ²=2.067 | 0.151 |
| 1 | 1538 (92.37) | 1069 (91.76) | 469 (93.80) |  |  |
| 2 | 127 (7.63) | 96 (8.24) | 31 (6.20) |  |  |
| APRI, N (%) |  |  |  | χ²=0.008 | 0.931 |
| 1 | 1063 (63.84) | 743 (63.78) | 320 (64.00) |  |  |
| 2 | 602 (36.16) | 422 (36.22) | 180 (36.00) |  |  |
| GPR, N (%) |  |  |  | χ²=2.262 | 0.133 |
| 1 | 1304 (78.32) | 924 (79.31) | 380 (76.00) |  |  |
| 2 | 361 (21.68) | 241 (20.69) | 120 (24.00) |  |  |

Table 2 Distribution of 7 Dichotomous Predictors in the Bleeding and Non-Bleeding Groups(Training Set)

| Variables | 0 (n = 1020) | 1 (n = 145) |
| --- | --- | --- |
|  |  |  |
| Post Liver Transplantation, n(%) |  |  |
| 1 | 975 (95.59) | 142 (97.93) |
| 2 | 45 (4.41) | 3 (2.07) |
| Pre-Corticosteroid, n(%) |  |  |
| 1 | 942 (92.35) | 122 (84.14) |
| 2 | 78 (7.65) | 23 (15.86) |
| Needle Depth, n(%) |  |  |
| 1 | 513 (50.29) | 93 (64.14) |
| 2 | 507 (49.71) | 52 (35.86) |
| PT, n(%) |  |  |
| 1 | 903 (88.53) | 112 (77.24) |
| 2 | 117 (11.47) | 33 (22.76) |
| ALT, n(%) |  |  |
| 1 | 299 (29.31) | 57 (39.31) |
| 2 | 721 (70.69) | 88 (60.69) |
| PLT, n(%) |  |  |
| 1 | 163 (15.98) | 56 (38.62) |
| 2 | 857 (84.02) | 89 (61.38) |
| GPR, n(%) |  |  |
| 1 | 833 (81.67) | 91 (62.76) |
| 2 | 187 (18.33) | 54 (37.24) |
